# Supplementary material for: Hierarchical rupture growth evidenced by the initial seismic waveforms
Source: Nat Commun. 2018 Sep 13;9:3714. doi: 10.1038/s41467-018-06168-3 (PMC6137102; doi:10.1038/s41467-018-06168-3)
Supplement: Supplementary file 1 — Supplementary Information [file 41467_2018_6168_MOESM1_ESM.pdf]

Hierarchical rupture growth evidenced by the initial seismic waveforms

Okuda et al.

Supplementary material

Supplementary Table 1. Earthquakes in the Naka region, as analysed in this study.

Group[a]	YY[b,c]	MM[c]	DD[c]	HH[c]	Latitude (°N)	Longitude (°E	Depth (km)	Magnitude (*M*)
C	2	9	21	22	36.425	140.688	51.2	1.6
other	2	10	13	4	36.432	140.688	51.4	3.9
other	2	10	13	5	36.430	140.687	51.5	1.6
other	3	1	1	23	36.426	140.656	52.6	3.6
other	3	2	6	21	36.411	140.681	49.5	1.9
other	3	4	25	11	36.428	140.690	51.4	1.6
other	3	5	11	2	36.431	140.686	51.5	2.3
M	3	6	9	18	36.427	140.689	51.3	4.7
other	3	6	19	8	36.425	140.661	52.5	3.3
other	4	1	3	3	36.431	140.685	51.6	3.8
other	4	2	29	10	36.417	140.667	50.9	2.7
other	5	2	1	22	36.427	140.681	48.4	2.0
other	5	4	9	4	36.428	140.664	52.4	2.4
C	5	5	19	4	36.425	140.688	51.3	1.6
C	6	9	24	13	36.425	140.688	51.3	1.9
other	7	1	4	0	36.431	140.689	51.4	4.2
A	7	5	18	17	36.427	140.686	51.5	3.9
other	7	5	25	15	36.458	140.694	56.4	1.7
B	7	6	3	6	36.425	140.689	51.3	2.9
M	7	11	30	18	36.425	140.689	51.3	4.7
other	8	8	13	10	36.432	140.685	51.6	3.8
other	9	4	6	19	36.430	140.686	51.5	1.7
other	10	2	9	12	36.428	140.690	51.3	1.6
other	10	3	3	20	36.450	140.666	49.6	1.7
A	10	4	29	7	36.427	140.686	51.4	4.2
other	10	5	7	4	36.431	140.688	51.4	3.9
other	10	12	5	6	36.414	140.688	51.0	3.5
other	11	3	14	15	36.464	140.730	51.7	4.2
other	11	3	26	14	36.431	140.685	51.6	3.7
other	11	5	25	0	36.424	140.698	51.7	3.1
B	11	7	10	18	36.426	140.688	51.4	3.2
other	11	7	24	19	36.430	140.687	51.5	2.2
B	11	8	29	12	36.424	140.689	51.3	2.9

M	11	9	10	15	36.425	140.688	51.4	4.8
other	11	10	4	18	36.429	140.662	52.5	2.1
other	11	10	10	6	36.415	140.688	51.0	1.7
other	12	3	9	14	36.430	140.688	51.6	1.7
other	12	7	28	15	36.416	140.688	51.1	1.7
other	12	8	1	13	36.425	140.661	52.4	3.5
other	12	9	4	16	36.418	140.679	51.3	2.0
A	12	12	6	19	36.426	140.685	51.4	4.3
C	12	12	15	10	36.425	140.688	51.3	2.1
other	13	5	30	1	36.430	140.686	51.6	3.6
other	13	9	10	16	36.428	140.661	52.5	3.0
M	13	10	12	2	36.425	140.691	51.3	4.8
other	13	10	13	19	36.430	140.686	51.5	2.5
other	14	3	10	1	36.442	140.686	52.1	2.3
other	14	3	29	1	36.414	140.688	51.0	1.8
other	14	4	25	8	36.431	140.687	51.5	4.1
other	14	7	1	0	36.416	140.688	51.1	1.7
other	14	12	20	9	36.430	140.686	51.6	1.8
C	15	5	11	20	36.425	140.688	51.3	1.9
other	15	8	3	3	36.414	140.689	51.0	1.7
other	15	9	19	14	36.423	140.695	51.0	2.2
other	15	11	15	8	36.428	140.690	51.3	1.6
M	15	11	22	8	36.426	140.687	51.4	4.8
other	16	5	22	2	36.415	140.688	51.1	1.6
other	16	12	19	16	36.431	140.688	51.5	3.8
C	17	2	17	18	36.425	140.688	51.3	1.8
other	17	5	4	22	36.430	140.686	51.6	1.8
other	17	6	1	15	36.416	140.688	51.1	1.7

[a]See the main text for a detailed description of the earthquake groups (M, A, B and C).

[b]"YY" represents the year after 2000.

[c]Date/time information is in UTC.
